# Variations in strain affect friction and microstructure evolution in copper under a reciprocating tribological load

**DOI:** 10.1557/s43578-020-00050-z

**Published:** 2021-01-25

**Authors:** Sarah Becker, Katrin Schulz, Dennis Scherhaufer, Peter Gumbsch, Christian Greiner

**Affiliations:** 1 Institute for Applied Materials (IAM), Karlsruhe Institute of Technology (KIT), Kaiserstrasse 12, 76131 Karlsruhe, Germany; 2 Institute for Micro Process Engineering (IMVT), Karlsruhe Institute of Technology (KIT), Hermann-von-Helmholtz-Platz 1, 76344 Eggenstein-Leopoldshafen, Germany; 3 Fraunhofer Institute for Mechanics of Materials (IWM), Woehlerstrasse 11, 79108 Freiburg, Germany; 4 KIT IAM-CMS MikroTribologie Centrum (µTC), Strasse am Forum 5, 76131 Karlsruhe, Germany; 5 Institute of Applied Research (IAF), Karlsruhe University of Applied Sciences, Moltkestrass 30, 76133 Karlsruhe, Germany

**Keywords:** Tribology, Copper, Strain distribution, EBSD, Microstructure evolution

## Abstract

**Abstract:**

The microstructure of the materials constituting a metallic frictional contact strongly influences tribological performance. Being able to tailor friction and wear is challenging due to the complex microstructure evolution associated with tribological loading. Here, we investigate the effect of the strain distribution on these processes. High-purity copper plates were morphologically surface textured with two parallel rectangles—referred to as membranes—over the entire sample length by micro-milling. By keeping the width of these membranes constant and only varying their height, reciprocating tribological loading against sapphire discs resulted in different elastic and plastic strains. Finite element simulations were carried out to evaluate the strain distribution in the membranes. It was found that the maximum elastic strain increases with decreasing membrane stiffness. The coefficient of friction decreases with increasing membrane aspect ratio. By analyzing the microstructure and local crystallographic orientation, we found that both show less change with decreasing membrane stiffness.

**Graphic abstract:**

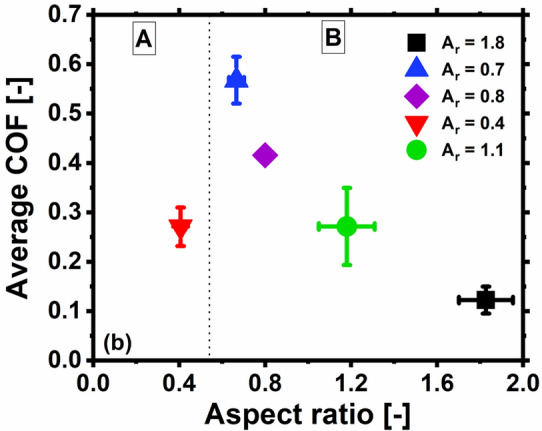

**Supplementary information:**

The online version contains supplementary material available at 10.1557/s43578-020-00050-z.

## Introduction

Correlating the microstructure of a material with its properties is one of the most central questions in materials science and engineering. Tribology is defined as the science of interacting surfaces in relative motion [1]. Tribological contacts are important for all machinery with moving parts from micro-electromechanical systems [2], via bionic [3] or medical applications like hip joints [4, 5] to the automotive industry [6]. Leonardo da Vinci and Guillaume Amontons defined the proportionality of an applied normal load to a measured friction force, thereby introducing the coefficient of friction (COF) [7]. Bowden and Tabor found that during a tribological contact of two metallic bodies, junctions are formed and sheared [7, 8]. The asperities of the harder material plough through the softer surface. Therefore, the properties of the softer material determine the friction and wear properties of the entire tribosystem [8]. The coefficient of friction of a tribological system can therefore be calculated by the ratio of the shear stress and the yield pressure of the softer material [7, 8]. This result demonstrates the relation between tribological behavior and material properties and therefore also the materials’ microstructure [9].

The first period in the life of a tribosystem is known as ‘running-in.’ Here, a mechanically altered layer grows from the contacting surface into the material [10]. This layer often has nanocrystalline or ultrafine-crystalline character [4, 11] and is referred to as “tribomaterial,” “third body,” or “tribolayer” [11]. This part of the subsurface microstructure can be different in crystallographic or chemical structure compared to the bulk [11–14]. Mostly, there is a sharp transition between the bulk material and the “tribolayer” where a change in microstructure occurs [13, 15, 16]. The orientation of the subsurface grains often is parallel to the direction of shear loading [17–21]. Due to the complexity inherent to all tribological systems, there are many parameters which have a significant effect on these microstructural changes. Among the principle ones are normal load and sliding speed, resulting in different stress and strain fields and thereby influencing the plastic response of the material [15, 16, 22]. Experiments have shown that the majority of the plastic deformation during sliding is carried by dislocation motion [23], resulting in an increase in dislocation density, a decrease in grain size, and a change in grain orientation [18, 24–26]. Numerical studies of different friction systems have investigated the effect of the sliding speed on the microstructure development under a tribological load [27, 28]. In addition, molecular dynamics simulations were applied to determine the influence of the stress field on the mechanisms for grain growth [29] or material transfer of the counter parts [30]. Dislocation dynamics simulations revealed the dislocation motion under the contact [31, 32]. The results demonstrated that dislocations are generated beneath the surface due to the shear stresses. The density of dislocations depends on the coefficient of friction. The simulations revealed that the maximum shear stress occurred in a shear band across the asperities [32]. Additionally, the strain distribution plays an important role in how the microstructure changes under a tribological load. With small strains, dislocation cells or walls form under the contact, separating coarse grains from smaller ones [33]. For high strain rate sliding, the grain refinement includes the formation of high dislocation densities near the surface, the presence of twins, dividing the coarse grains into smaller nano-sized ones [22, 34].

Based on the experimental and numerical results present in the literature, the complexity of the microstructural changes under a tribological load becomes apparent. The elementary mechanisms acting and dominating under different experimental conditions need to be studied in order to be able to strategically influence these complex processes. Our own recent work, for example, has identified three processes acting in the very early stages of sliding [35]: a simple shear of the subsurface area in sliding direction, a highly localized shear in sliding direction at a dislocation self-organization feature called the dislocation trace line, as well as a crystal rotation of the material between the sample surface and the dislocation trace line around an axes parallel to the transvers direction [13]. In light of these and other results, either by this group of authors or others, it is so far not fully understood how different elastic and plastic strains influence the subsurface microstructure evolution. In order to investigate this problem, rectangular copper structures—referred to as membranes throughout the rest of the manuscript—were created by micro-milling and with different aspect ratios; the aspect ration being defined as the membrane height divided by its thickness. Copper is a widely used face centered model material and ideally suited for our experimental approach. The membrane structures allowed to systematically alter the strain in the material. The resulting microstructures were examined by electron microscopy. Once we understand the effect of the strain distribution in such a simplified model system, we might be able to transfer this knowledge to other materials and more complex and realistic tribological contacts.

## Results

### Finite element simulations

**Figure 1:**
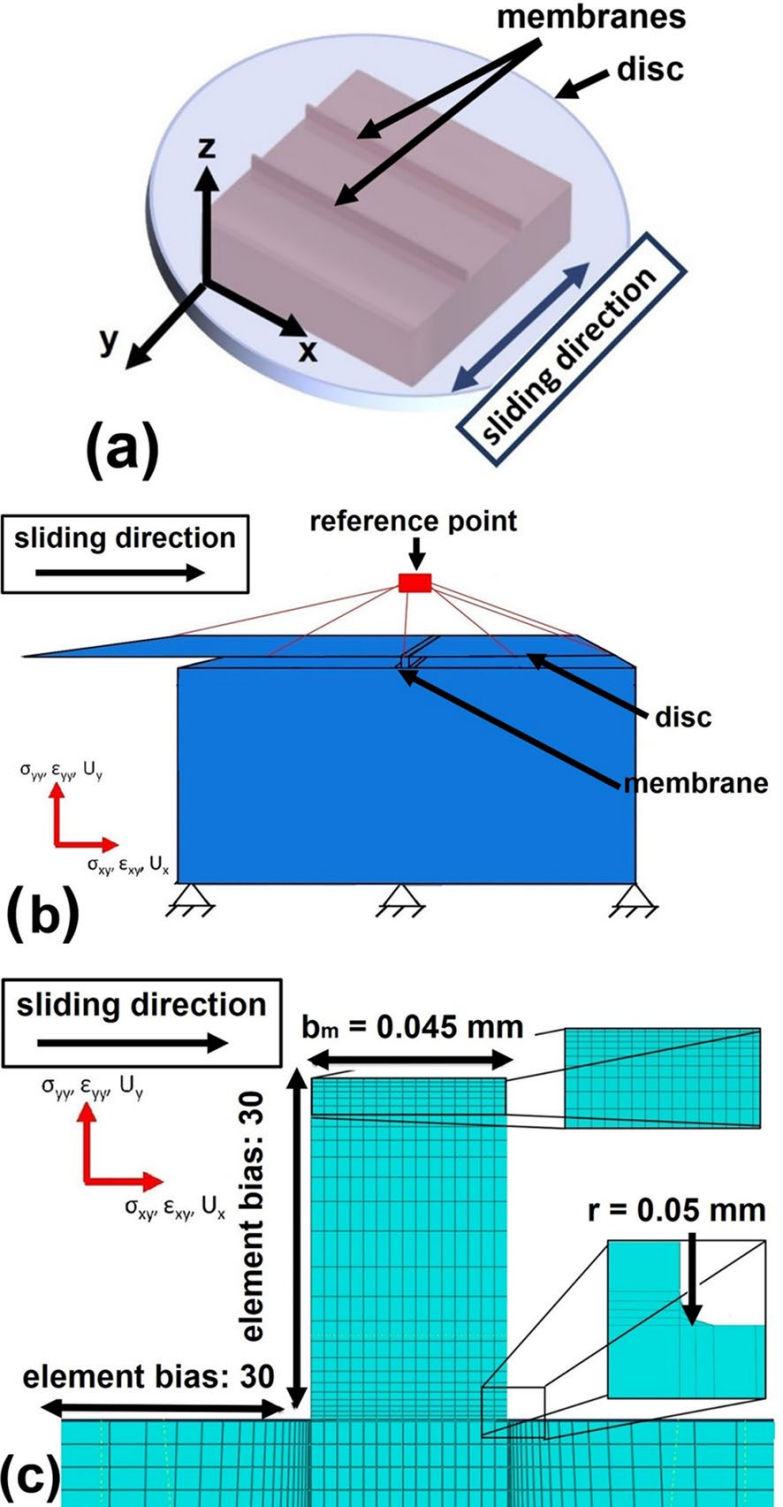
Schematic of the contact partners and finite element model for the contact situation. (a) Membrane sample and sapphire disc. The disc moves along the *y*-axis, perpendicular to the membranes. (b) Boundary conditions and sliding direction. The disc moves in positive *x*-direction, perpendicular to the membrane. Only one membrane is modeled. The sliding distance is Δ*x* = 0.05 mm. Above the sample, a reference point is located. The bottom of the sample is fixed. (c) Finite element network with the area beneath the contact and the edges. These elements have a finer mesh and the edges are rounded. Both (b) and (c) are side views of the sample in touch with the sapphire disc.

**Figure 2:**
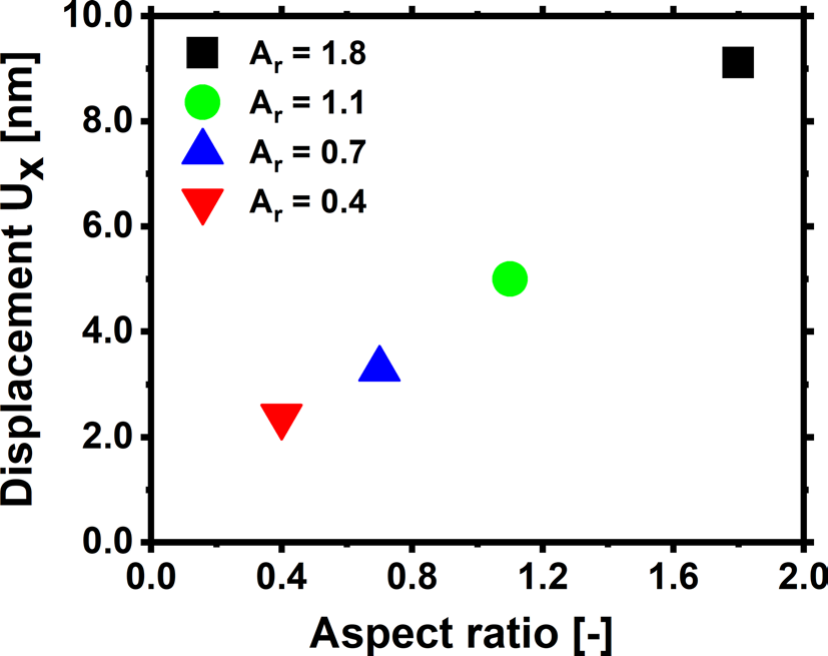
FEM results for the displacement of the membranes in x-direction as a function of the aspect ratio.

**Figure 3:**
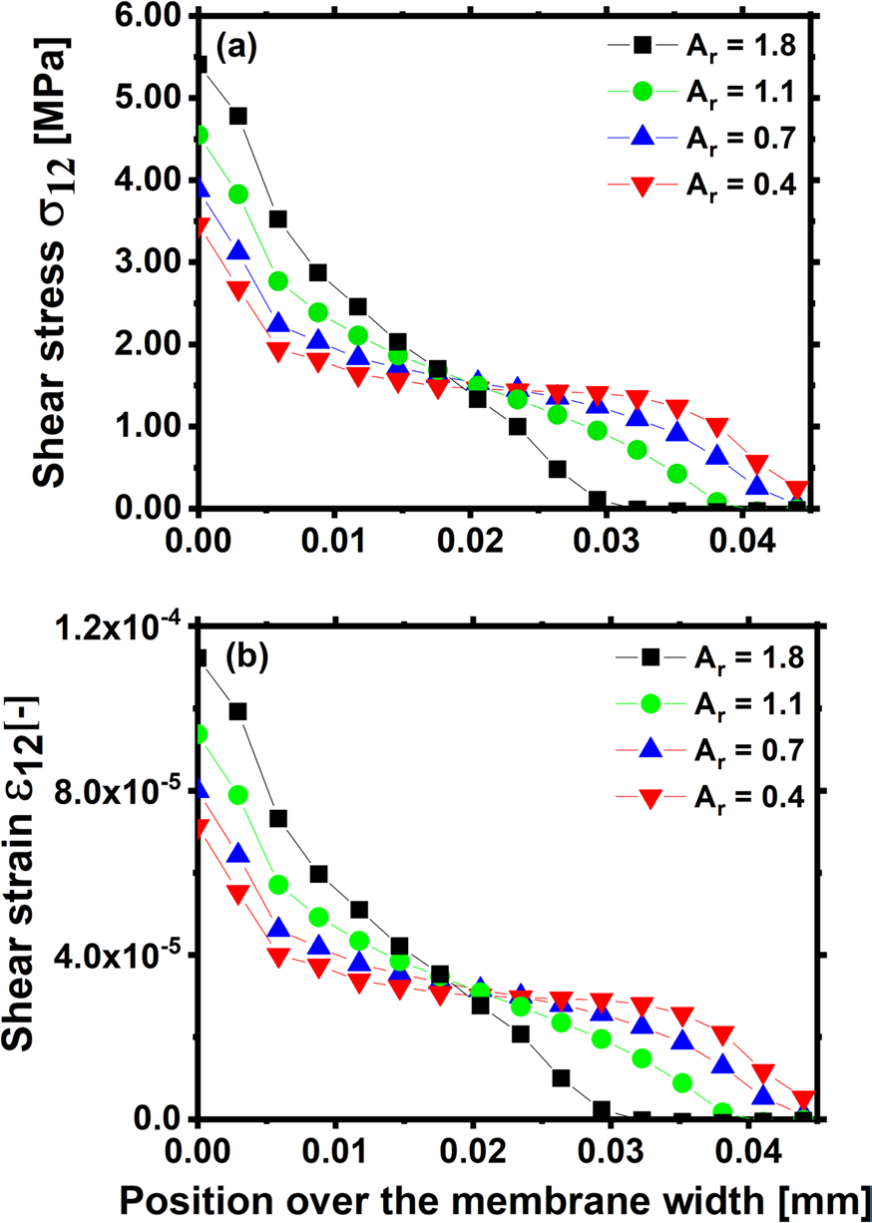
Shear stress (a) and shear strain (b) distribution according to the FEM model directly under contact surface for all four aspect ratios. The coefficient of friction is set to COF = 0.3 for all aspect ratios; the sliding distance 0.05 mm and the applied normal force 2 N.

In order to assess the stresses in the membranes during the tribological experiments, finite element simulations for each aspect ratio were conducted. The aspect ratio A_r_ is thereby defined as the membrane height divided by the membrane thickness. See Fig. 1a for a schematic drawing of the contact situation and details of the finite element model which was applied to calculate shear stress and strain. First, the displacements of the membranes were simulated and evaluated. Figure 2 shows the displacement in x-direction for all aspect ratios. The plot illustrates that the displacement of the membranes increases with the aspect ratio. The displacement measured for the membranes with an aspect ratio of *A*_r_ = 1.8 is a factor of 3.8 higher than for *A*_r_ = 0.4. Figure 3b presents the shear strain distribution over the width of the membranes for all four aspect ratios. While the disc is moving to the right, the highest shear stresses occur at the left edge of the membrane; the stress decreases along the width. Analyzing the distribution along the width for membranes with an aspect ratio *A*_r_ = 1.8, it becomes apparent that the strain reaches zero on the membrane surface at 35 µm distance from the membrane’s left edge. This position moves to the right as the aspect ratio decreases. The shear stress at the left edge decreases with decreasing aspect ratio. The maximum shear strain *ε*_12_ is found for the membranes with an aspect ratio *A*_r_ = 1.8. The minimum shear strain occurs for the membranes of *A*_r_ = 0.4.

### Tribological properties

**Figure 4:**
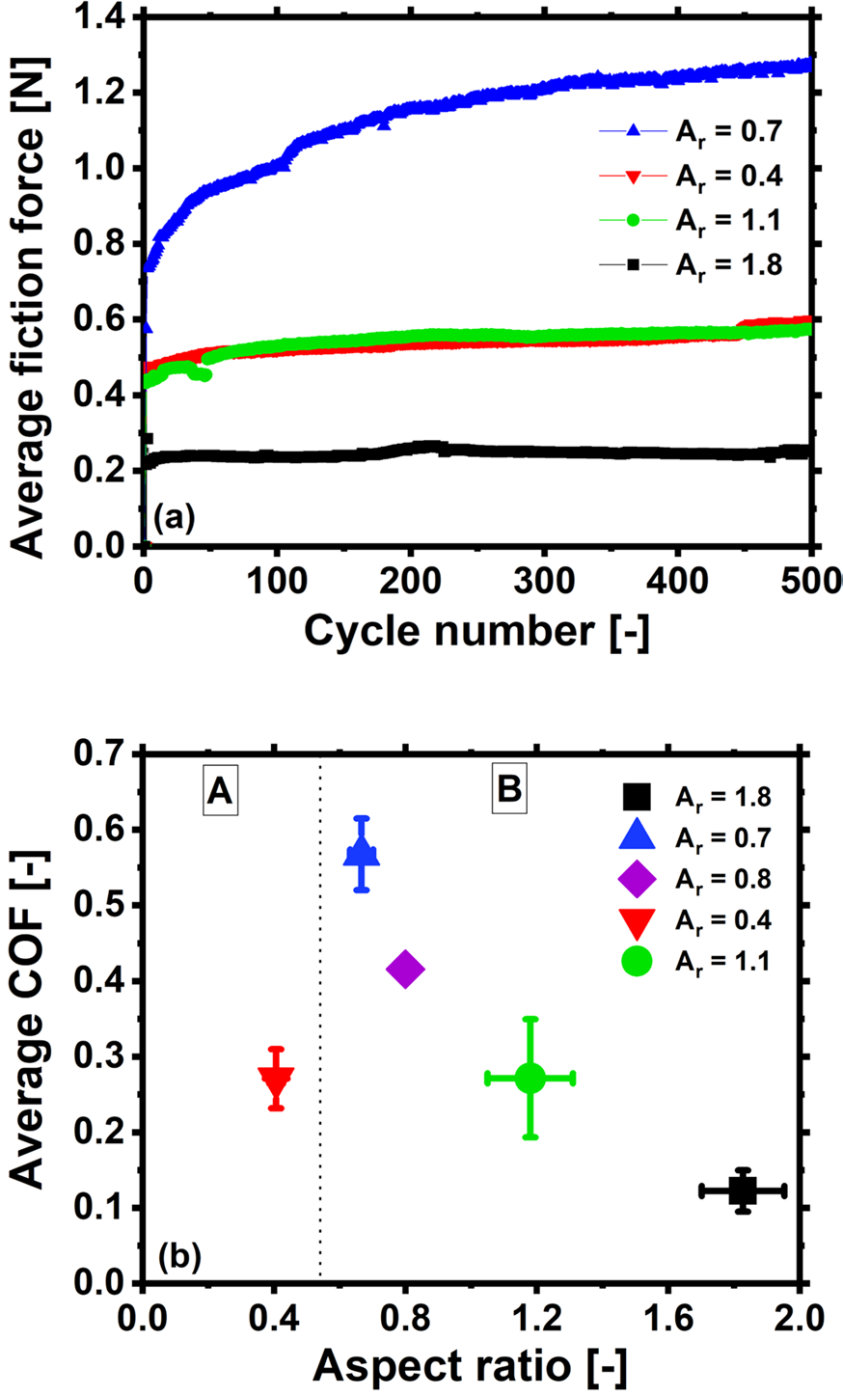
Experimental friction force and coefficient of friction averaged over 500 cycles as a function of aspect ratio. (a) The tests were run for up to 500 cycles. (b) The figure is separated into two regions: Region A for *A*_r_ = 0.4 and Region B containing the membranes with aspect ratios *A*_r_ = 0.7 to *A*_r_ = 1.8.

In order to characterize the tribological behavior as a function of the membranes’ aspect ratio, the friction force is plotted for 500 cycles and all four aspect ratios in Fig. 4a. The friction force increases with decreasing aspect ratio, with the exception of the membranes with an aspect ratio of *A*_r_ = 0.4. Figure 4b presents the value of the COF averaged over all 500 cycles. The figure is separated into two regions: Region A represents the membranes with an aspect ratio of *A*_r_ = 0.4. For these samples, COF is *µ* = 0.29. Region B contains the membranes with an aspect ratio of *A*_r_ = 0.7 to 1.8. For membranes with the aspect ratio *A*_r_ = 0.7, an COF of *µ* = 0.55 was measured. This simultaneously represents the maximum of all measured values. The coefficient of friction in region B decreases with increasing membrane aspect ratio.

### Membrane surfaces

**Figure 5:**
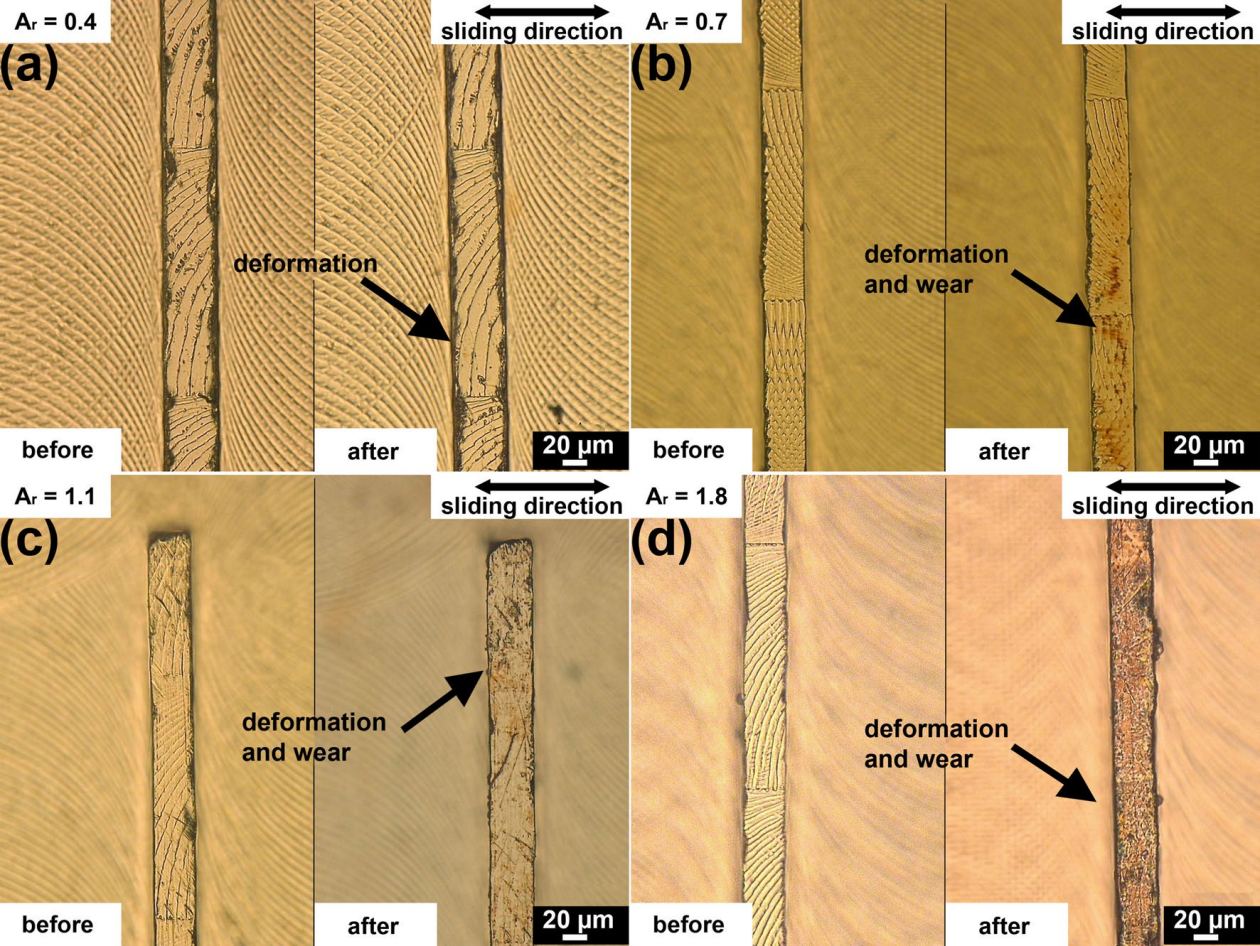
Optical microscopy images for membranes with different aspect ratios before and after reciprocating tribological loading. (a) *A*_r_ = 0.4; (b) *A*_r_ = 0.7; (c) *A*_r_ = 1.1; and (d) *A*_r_ = 1.8. In each panel, the image on the left is from a membrane before a test and the right one after. Characteristic surface changes are marked by black arrows.

To follow possible changes on the membranes’ contact surfaces, Fig. 5 presents optical microscopy images before and after performing the experiments for the membranes of the aspect ratios (a) *A*_r_ = 0.4; (b) *A*_r_ = 0.7; (c) *A*_r_ = 1.1 and (d) *A*_r_ = 1.8. To retrieve the same position on each membrane surfaces before and after the experiment, milling lines were used as fiducials. For membranes with an aspect ratio *A*_r_ = 1.8 and after 500 cycles, the milling lines were no longer visible, instead a brown colored surface layer is observed. This color is darker than it would be for native copper. By comparing the surfaces of different membranes, it becomes apparent that for the membranes with smaller aspect ratios, less changes are observed on the contacting surfaces (Fig. 5a, b).

**Figure 6:**
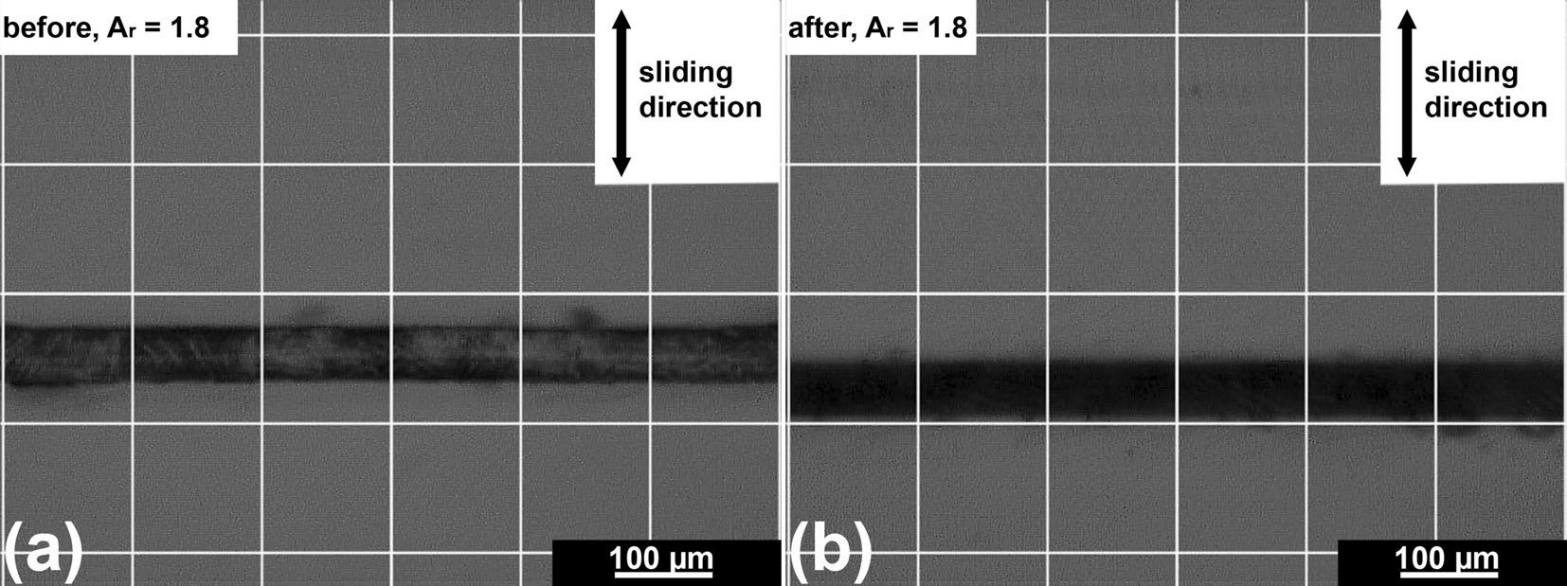
Images of a membrane with an aspect ratio *A*_r_ = 1.8 taken through the sapphire disc by the high-speed camera investigating the experiments in situ. Both images are taken at the upper dead center; in (a) after the first cycle and in (b) after 500 cycles.

These results are confirmed by images acquired with the high-speed camera. At every dead center, the high-speed camera captured the membranes’ surfaces. Figure 6 shows the results for the membranes with the highest aspect ratio (*A*_r_ = 1.8) before and after 500 cycles. Here, again a change on the surface occurred and after the tribological loading, the milling lines are no longer visible.

**Figure 7:**
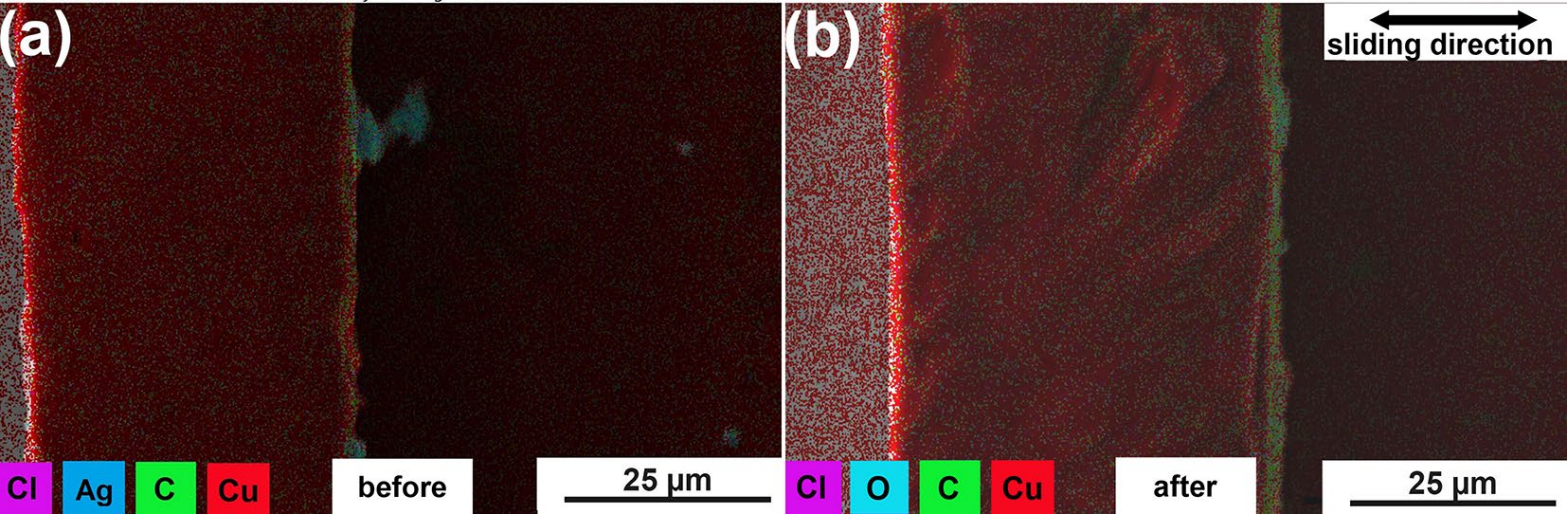
EDS mappings (a) before and (b) after an experiment (500 cycles) for a membrane with an aspect ratio of A_*r*_ = 1.8.

In order to analyze any chemical changes on the membranes’ surfaces, EDS measurements before and after an experiment were performed. Figure 7 presents the results of these analyses. Before the experiment, we measured a composition of 98.7 wt% copper, 0.2 wt% carbon, and 0.7 wt% silver (originating from the adhesive used during electron microscopy). After an experiment (Fig. 7b), additional 1.1 wt% of oxygen was detected, compared to 95.8 wt% copper and 3.0 wt% carbon.

### Microstructure evolution in the membranes

**Figure 8:**
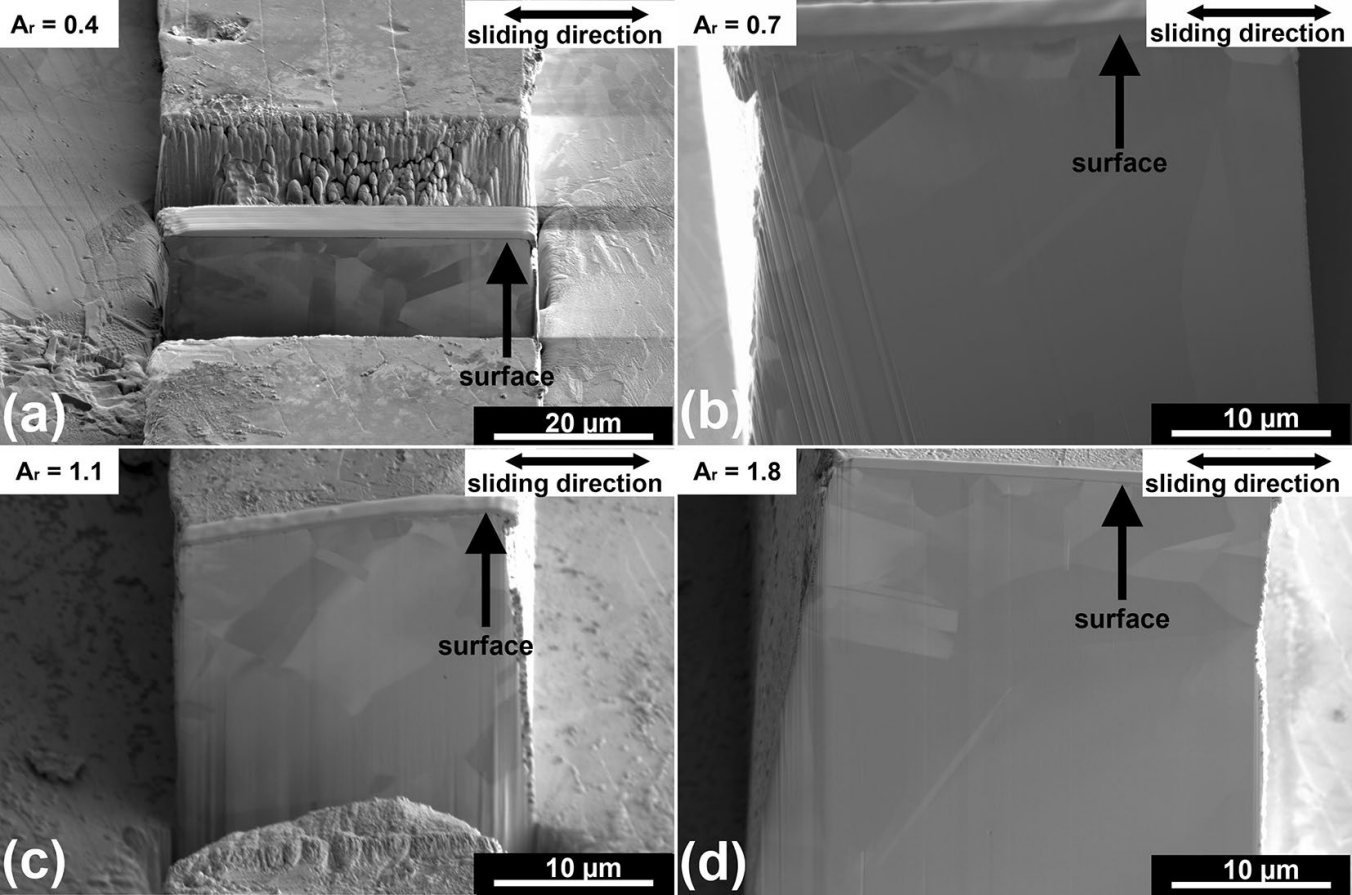
Scanning electron microscopy (SEM) images of membranes with different aspect ratios after 500 cycles of tribological loading. (a) *A*_r_ = 0.4; (b) *A*_r_ = 0.7; (c) *A*_r_ = 1.1 and (d) *A*_r_ = 1.8. The cross-sections were performed on the entire cross-sectional area of the membranes, perpendicular to the sliding surface and parallel to the sliding direction. The contrast at the top of the images is from the two protective platinum layers; the copper surface is marked by arrows.

To investigate the microstructure evolution, FIB cross-sections were prepared in the middle of the membranes after 500 cycles, parallel to the sliding direction. In the unloaded material (Fig. S1), neither a contrast change nor an altered microstructure in the subsurface area of the membranes was observed by electron microscopy. After tribological loading (Fig. 8), a modification in the subsurface area was found. Edge rounding is observed in membranes with higher aspect ratios. The FIB cross-section for an aspect ratio of *A*_r_ = 0.4 (Fig. 8a) demonstrates that this change occurred over the whole membrane width. Figure 8b presents the FIB cross-section for an aspect ratio of *A*_r_ = 0.7. Beneath the surface, a slightly changed microstructure is detected in the form of a tribologically deformed layer. With further distance from the contact surface, the material appears to be unchanged. FIB cross-sections for *A*_r_ = 1.1 (Fig. 8c) do not show significant changes in the microstructure beneath the surface. In addition, there is no evolution of the microstructure at the membrane’s bottom. Figure 8d presents a FIB cross-section for *A*_r_ = 1.8. Directly beneath the surface a slight contrast change compared to the bulk material can be observed. In the bottom half of the membrane, these changes are no longer detected.

### Cross-sectional electron backscatter diffraction mapping

**Figure 9:**
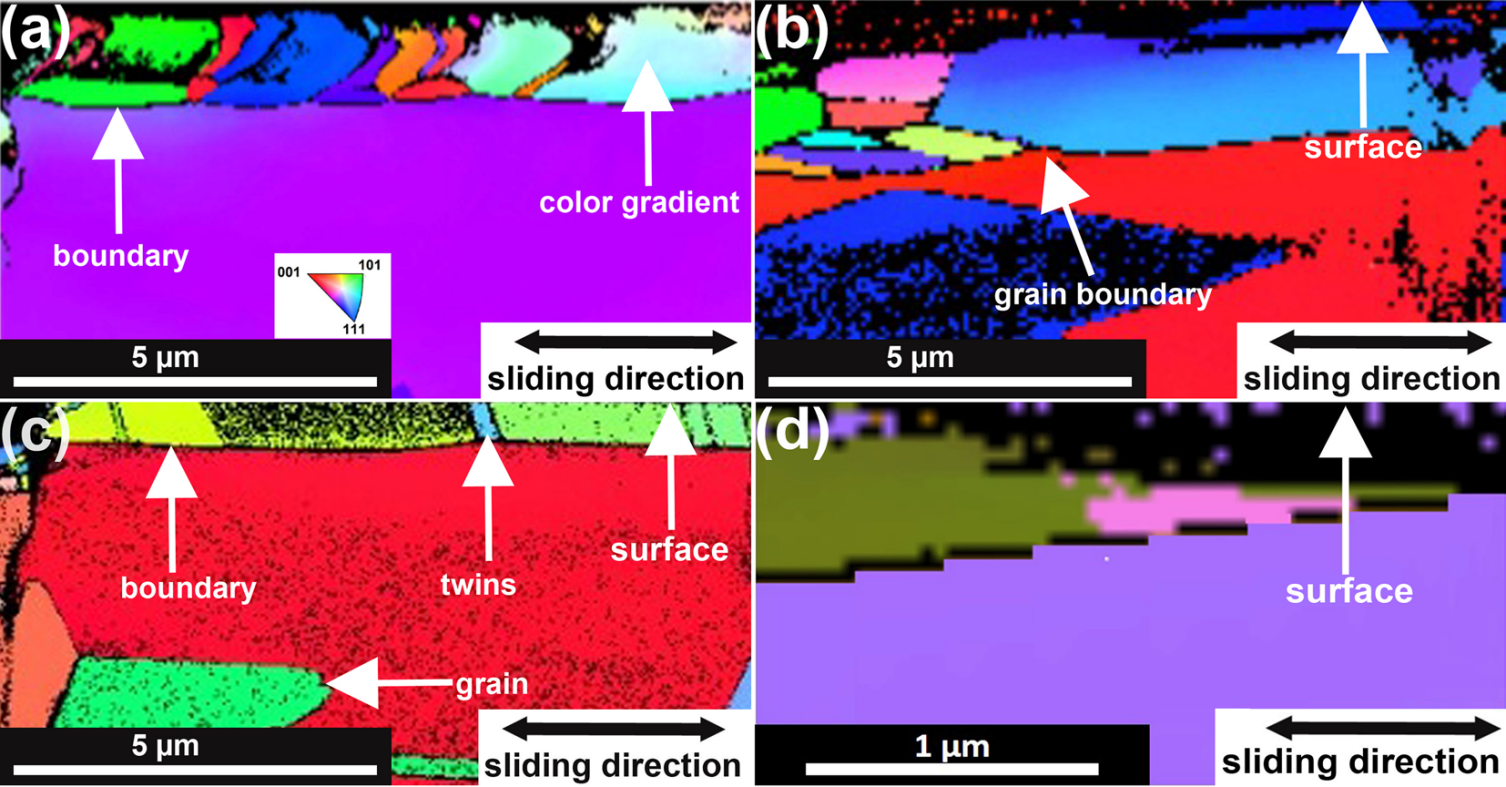
Orientation maps obtained from cross-sectional electron backscatter diffraction performed on membranes with different aspect ratios after 500 cycles of tribological loading. (a) *A*_r_ = 0.4; (b) *A*_r_ = 0.7; (c) *A*_r_ = 1.1; and (d) *A*_r_ = 1.8 are presented. The maps are given with respect to the transvers direction. The inset in (a) shows the color code of the inverse pole figure. Important features are marked by arrows.

To determine the possible changes in crystallographic orientation and to gain information on the density of geometrically necessary dislocations (GNDs) due to tribological loading, EBSD measurements were taken on membrane cross-sections. See Fig. 9 for the orientation data for all four membrane aspect ratios and with respect to the normal direction (ND). For all four aspect ratios, we find a boundary nearly parallel to the surface which separates the bulk grains and a near-surface area. The smallest aspect ratio (*A*_r_ = 0.4) shows a decreased grain size under the contact and the grains are tilted roughly 45° to the sample surface. The orientation map demonstrates a color gradient inside one grain (marked by the white arrows in Fig. 9a. There is a sharp transition between the deformed layer beneath the surface and the bulk material. The membrane with an aspect ratio *A*_r_ = 0.7 (Fig. 9b) shows a localized decrease in grain size at the right edge of the indexed area. No tilting of the grains is visible. For an aspect ratio of *A*_r_ = 1.1 (Fig. 9c), there is a sharp transition between a thin layer directly under the contact and the bulk material. In the deformed layer beneath the surface, twins are identified (marked by the white arrow). The orientation map for membranes with *A*_r_ = 1.8 (Fig. 9d) was measured at a higher magnification. No altered subsurface could be identified and a layer of which the crystallographic orientation can hardly be determined is found directly beneath the surface.

**Figure 10:**
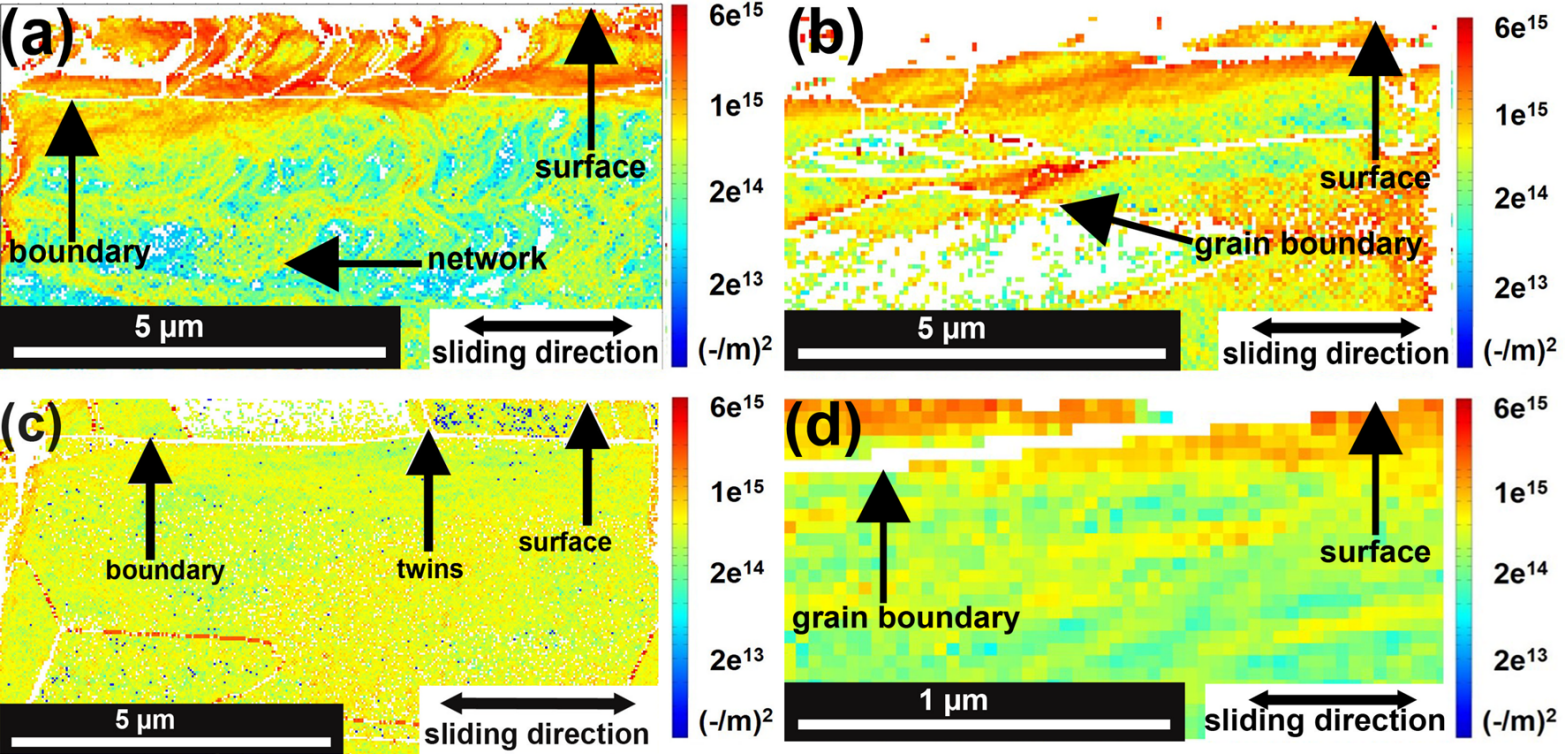
Geometrically necessary dislocation (GND) density in the cross-sectional area of the membranes after 500 cycles of tribological loading. (a) *A*_r_ = 0.4; (b) *A*_r_ = 0.7; (c) *A*_r_ = 1.1; and (d) *A*_r_ = 1.8. The samples’ surfaces are marked by black arrows. The color bar in the GND density mapping represents the number of GNDs per unit area (m^2^).

Crystallographic orientation gradients can be interpreted as the GND density. Performing such an analysis—as outlined in more detail elsewhere [13, 36]—leads to the results presented in Fig. 10. With increasing membrane aspect ratio, the average GND density decreases beneath the contact area (*ρ*_GND_ = 6 × 10^15^ m^−2^ for *A*_r_ = 0.4 to *ρ*_GND_ = 2 × 10^14^ m^−2^ for *A*_r_ = 1.8. This indicates a heavy plastic deformation in and under the contact area due to the tribological load. The plastic deformation decreases into the bulk.

## Discussion

### Contact character

The results of the finite element simulation show that the contact area of the membranes is characterized by a stress and strain gradient along the contact surface. When the sapphire disc moves from left to right, the maximum absolute value of the shear stress is found at the upper left edge of the membranes and vice versa on the retrace of the disc at the upper right edge. The shear stress gradient increases with membranes aspect ratio (Fig. 3). For higher aspect ratios, the shear stress reaches zero for a portion over the membrane width. Consequently, the stresses act on a smaller and smaller contact area as the aspect ratio increases. One should note, however, that imperfections of the real contact, such as surface roughness and a (even small) misalignment between the sapphire disc and the membranes, might alter these results. The same is true for edge rounding which was not taking into account in our FEM analyses. At the same time and not surprisingly, the FEM results do show that with increasing aspect ratio the stiffness of the membranes decreases and the lateral displacement increases. Both have an effect on the stress and strain distribution. It is evident from simple mechanics that a higher membrane has a larger lever arm and a lower resistance moment [37, 38]. More interestingly, once contact between disc and sample has been established and the material starts to slide, on the left edge of the membranes a torque arises. However, the membranes cannot tilt due to the flat-on-flat contact with the disc and the applied normal load. Therefore, the contact stresses are highest on the left edge of the membrane, when the disc moves to the right (see Figs. S3 and S4). The area of the contact where the shear stress is non-zero is referred to as the “effective surface” for the remainder of the manuscript. With decreasing membranes' aspect ratio, this effective surface increases.

Due to these differences on effective area and maximum shear stresses, the friction behavior of the membranes is separated into two regions as depicted in Fig. 4b. In Region A and for *A*_r_ = 0.4, the higher stiffness of the membranes causes a uniform load distribution, the largest effective surface and thereby small differences in stresses in the contact area. These samples therefore behaved like bulk copper. Also the measured friction coefficient is very similar to literature values for high-purity copper tested in dry sliding against sapphire [36].

In Regime B and for aspect ratios *A*_r_ = 0.7 to 1.8, the coefficient of friction decreases with increasing aspect ratio (Fig. 4b). As the membranes get more compliant with increasing aspect ratio, the effective surface is reduced, and the lateral displacement increases. The load configuration can therefore no longer be compared to a tribological test on a bulk sample but now comprises a combination of friction and elastic shearing as well as supposedly also some plastic shearing. The latter fraction increases with aspect ratio, resulting in the observed decrease in friction coefficient.

This effect of membrane aspect ratio also explains why for an aspect ratio of *A*_r_ = 1.8 , the highest amount of loose wear particles was detected after 500 cycles of loading. Here, the highest torque acting on the membrane’s edge has resulted in wear. This is in line with the optical micrographs for all four aspect ratios presented in Figs. 5 and 6 and the EDS mapping in Fig. 7. EDS revealed an increase in oxygen content after the experiment which most probably is due to tribochemically assisted oxidation [14, 39].

At the same time, we cannot rule out that for the lowest aspect ratio, a longer running-in period might be observed so that at one point the friction coefficient would start to increase. The same is true for the onset of tribo-oxidation which certainly will start to become a factor influencing friction and wear behavior [36]. One could also argue that the *A*_r_ = 0.4 samples show a steady-state friction behavior as here the contact stresses are below a certain threshold value for a different wear mechanism, compared to the larger aspect ratio membranes. One might also wonder about different peak surface stresses as a function of membrane aspect ratio. Calculating these stresses most likely requires a more in depth contact mechanics analyses and is beyond the scope of this paper. As for why the *A*_r_ = 0.7 sample shows the longest running-in period, we can only speculate that the apparent change in mechanism when crossing from regime A into regime B (Fig. 4b) causes this phenomenon.

### Subsurface microstructure

These considerations regarding the stress and strain distribution are consistent with the subsurface microstructures as revealed by FIB cuts (Fig. 8) and cross-sectional EBSD (Figs. 9 and 10). In a nutshell, the combination of lower dimensional stability, higher stresses and strains at the edges, results in less extended and more localized microstructural changes with increasing aspect ratio.

Discussing these results in a bit more detail, literature teaches us that microstructural observations as those made in Fig. 8 are most like associated with subsurface plastic deformation carried by dislocation motion [12, 13]. For membranes with *A*_r_ = 0.7, a grain refinement near the surface is found and EBSD (Fig. 9) reveals different grain orientations. In some instances, orientation gradients are observed (marked by an arrow). In contrast, the subsurface grain size within the membranes with aspect ratios *A*_r_ = 1.1 and *A*_r_ = 1.8 (Fig. 8c, d) did not show any direct evidence of microstructural changes or severe plastic deformation other than the boundary underneath the surface in Fig. 8c. In EBSD, for *A*_r_ = 1.1, a homogeneous subsurface layer with a thickness of approximately 800 nm becomes visible (Fig. 9c). In this layer, twins are observed. It is unlikely that these twin boundaries are due to heat treatment or recrystallization [40] and even though mechanical twinning is usually not observed in pure copper at room temperature [22, 35], due to the extreme shear conditions induced by the tribological load [41], these twins must most probably be attributed to the mechanical tribological deformation.

The horizontal nature of the grain boundary separating the near-surface layer from the bulk microstructure both in Fig. 9b and c reminds of the dislocation trace line phenomenon reported on for very low cycle numbers between pure copper and a sapphire sphere [13, 36]. This assumption is substantiated by the GND evaluation at least for *A*_r_ = 0.4 (Fig. 10a). The horizontal boundary confines a significant increase in GND density to the area above the horizontal line.

Generally, the GND density decreases with membrane aspect ratio, from *ρ*_GND_ = 6 × 10^15^ m^−2^ for *A*_r_ = 0.4 to *ρ*_GND_ = 2 × 10^14^ m^−2^ for *A*_r_ = 1.8. This suggests a decrease in plastic deformation with increasing membrane aspect ratio, which is consistent with the results presented above and the idea that as the aspect ratio increases, the decrease in bending stiffness leads to more elastic deformation.

For the membranes with an aspect ratio of *A*_r_ = 0.7, *A*_r_ = 1.1 and *A*_r_ = 1.8, these results can be understood together with the friction response: with increasing aspect ratio, their stiffness decreases and the mechanical load acts on a smaller and smaller effective surface. This results in less microstructural changes over the entire width of the membrane, but wear will increase. This is exactly what we observed in our experiments and also explains the edge rounding observed in membranes with higher aspect ratios (see Fig. 8). For the membranes of *A*_r_ = 0.4, there is significantly less torque acting at the membranes’ edges, leading to less wear and a more homogeneous—bulk-like—subsurface microstructure evolution. As mentioned above, this is also reflected in a friction coefficient that is similar to that of bulk high-purity copper when paired against sapphire.

We can only speculate why the grains in the subsurface area for *A*_r_ = 0.4 are at a preferential angle towards to sliding surface. In the literature, a similar phenomenon was described for pin-on-disc experiments [18, 19, 21, 42]. Even though these authors offer different reasons for a grain orienting and bending, the observation itself can easily be rationalized for such unidirectional sliding tests. Here, however, for a reciprocating motion, the explanation most probably be more intricate. Perhaps the original grain subjected to the tribological load (seen towards the bulk of the membrane in Fig. 10a) had one specific set of slip systems favorably oriented under this angle towards the contact surface. This could explain why here the dislocation density increases in a fashion allowing to form new (subgrain) grain boundaries.

## Conclusions

In this contribution, the effect of different elastic and plastic strains was systematically investigated by making use of morphologically textured samples. These consisted of membrane-like features in the form of two parallel rectangles with a length of 8 mm which where micro-milled into high-purity copper plates. The height of these membranes was varied and the width was kept constant at 45 µm. This resulted in samples with membrane aspect ratios between *A*_r_ = 0.4 and 1.8. These samples were paired against sapphire discs in reciprocating dry sliding experiments for 500 cycles at a stroke length of 500 µm and a normal load of 2 N. The ensuing subsurface microstructure evolution was monitored with dual-beam microscopy, STEM and EBSD. A finite element simulation was performed to reveal the strain distribution in the contact as a function of aspect ratio. We can summarize our results as follows:

- The membranes with *A*_r_ = 0.4 behave bulk-like. This goes for their friction coefficient as well as for the subsurface microstructure evolution.
- This is explained through their low aspect ratio which makes them less prone to elastic bending, thus accommodating the shear induced by the tribological load mainly by dislocation motion and self-organization.
- For membranes with *A*_r_ = 0.7, 1.1, and 1.8, their mechanical stiffness decreases with aspect ratio. Therefore, more and more of the tribological load is accommodated by elastic bending of the membrane.
- Due to the flat-on-flat contact configuration, this results in a decrease in the effective contact area with aspect ratio and a confinement of wear generation to the membrane edges.
- Consequently, the friction coefficient decreases with aspect ratio as well as there is less and less subsurface microstructural evolution but increased wear.

These results, albeit just a first step, demonstrate that a change in elastic strain for these membrane structures does have a significant effect on the coefficient of friction and the material itself.

## Material and methods

### Sample preparation

Oxygen-free high-purity (OFHC) copper plates (Goodfellow, Friedberg, Germany) with a purity higher than 99.95% and a hardness of 48 HV 0.1 (Fischerscope H100C Tester, Sindelfingen, Germany) were used. The copper plates were annealed in a vacuum of 1.5 × 10^−6^ mbar for 2 h at a temperature of 500 °C. For cooling down to room temperatures, the plates were left inside the furnace under vacuum (with an average cooling rate of 80 K/h). The annealing process was followed by structuring the samples. The plates were cut into pieces with a size of about 8 × 8 × 3 mm^3^ and afterwards structured by micro-milling. Two parallel rectangles over the entire sample length remained after milling (Fig. S1 of the Supplementary Information). By keeping the width of the membranes constant (*b*_m_ = 45 µm), the contact area and the nominal corresponding contact pressure (*P*_m_ = 2.8 MPa) were also constant for every experiment. Varying the height of the membranes (*h*_m_ = 20, 30, 50, 80 µm), resulted in aspect ratios of *A*_r_ = 0.4, 0.7, 1.1, and 1.8, as well as in different elastic and plastic strains during the tribological experiments. After structuring the samples, they were annealed with the same parameters as above. Hence, all deformation caused by machining was removed (see Fig. S2 for a scanning transmission electron microscopy images before and after the final heat treatment). The average grain size was determined to be between 30 and 40 µm. The samples were ultrasonically cleaned in isopropanol for 15 min right before the experiments. This sample preparation process was developed in order to achieve a comparable initial microstructure and reproducible surface chemistry for all membranes. Due to the heat treatments, the initial microstructure showed a homogenous and low defect density.

### Tribological testing

Sapphire discs (GWI Sapphire, Lauf, Germany) with a diameter of 50 mm were chosen as counter bodies in the tribological experiments for their extremely low surface roughness (root mean square roughness below 10 nm as measured by atomic force microscopy), high hardness, and optical transparency. Sapphire’s optical transparency was necessary for an in situ observation of the contact and the investigation of the surface development in the membranes using a high-speed camera [43]. The tribological experiments were performed in reciprocating motion under dry conditions, with 50% relative humidity and at room temperature. The only variable in the tests was the height of the membranes. All other parameters were kept constant. A normal load of 2 N was applied, the sliding speed was set to 500 µm/s. A stroke length of 500 µm was chosen. The surfaces of both membranes were in contact with the sapphire disc (Fig. 1a). Each sliding test was performed in a new region on the disc, so that the initial tribological contact in terms of roughness and purity was comparable for all experiments. During the tests, the normal load—applied by dead weights—and the friction force were measured by a 3-component force sensor (Kistler, Sindelfingen, Germany). At least two samples per aspect ratio were tested.

The contact character was investigated with a high-speed camera VW-9000 from Keyence (Osaka, Japan). Still images were taken at both dead centers up to 450 cycles. For these images, the camera was not used in high-speed mode. The images for each dead center were used to evaluate the development of the membrane’s surfaces. From 450 to the maximum cycle number of 500, the high-speed camera recorded videos of the contact area. The frame rate was 1000 fps at VGA resolution (640 × 480 pixels).

### Microstructure investigation

Focused ion beam/scanning electron dual-beam microscopy (FIB/SEM; Helios NanoLab DualBeam 650 from FEI, Hillsboro, OR, USA) was applied to monitor the microstructure evolution due to the tribological experiments. Each sample was first thoroughly investigated by optical means and again once the sample was inside the DualBeam FIB. Focused ion beam cross-sections were cut at locations that we deemed to be the most representative ones for each sample.

A FIB lift-out technique with little ion beam damage was used to prepare the scanning transmission electron microscopy (STEM) and electron backscatter diffraction (EBSD) foils for this study. Two deposited platinum layers protected the surface. The first one was applied within the FIB with the electron beam, the second thicker layer was deposited with the ion beam [44]. Scanning transmission electron microscopy images were taken with an acceleration voltage of 30 kV and beam current of 100 pA. Electron backscatter diffraction was used to determine the grain size and the grain orientation. Cross-sectional EBSD scans were performed on a 70° pre-tilted surface. Orientation maps were taken with an acceleration voltage of 25 kV or 30 kV and beam currents of 3.2 nA and 6.4 nA. A step size of 50 nm was chosen. The detector NordlysMax^2^ acquired the Kikuchi patterns and the software AZtecHKL (both, Oxford Instruments, Oxfordshire UK) was used for gathering the EBSD data [45]. From these data, misorientations can be interpreted as density of geometrically necessary dislocations (GND). The GND density was calculated with MTEX, a Matlab toolbox, as described earlier [13, 45–47]. The direct information in the EBSD results is the crystallographic orientation of each pixel, which can be used to calculate a crystallographic orientation gradient. In some instances, not the entire area could be completely indexed due to heavy plastic deformation. Furthermore, the protective platinum layers were also not indexed due to their amorphous nature.

### Finite element simulations

As the stress and strain distributions in the contacting materials are not experimentally accessible, a finite element simulation was used to define the stress and strain conditions in the contact area. The finite element program Abaqus (Dassault Systèmes, Paris, France) was employed. To simplify the model, only one membrane was taken into account. The bottom of the sample was fixed, while the membrane could move in the normal and tangential direction. The disc was constructed as a rigid body, with a significantly higher stiffness compared to the membrane. Initially, both surfaces are not in contact. At the beginning of the simulation, these are then brought into contact. In all simulations, the disc moved in positive x-direction, which means from left to right. The discs’ paths were *s* = 500 µm long. Above the sample, a reference point was defined, which applied and controlled the force and the path (Fig. 1b). Figure 1c presents the finite element mesh in more detail. In the areas where large deformations or stresses were expected, the mesh was refined by partitioning. Edge effects were reduced by rounding the edges. In total, the mesh had 30,500 eight-node brick elements. The FEM modeling was performed considering elastic deformation only. A Young’s modulus of 117 GPa for copper and of 345 GPa for sapphire was used; the Poisson ratios were 0.34 and 0.3, respectively. The normal and tangential loads were applied in a two-step process. First, the membrane was loaded with a normal force of *F*_N_ = 2 N. The force was applied linearly over the above-mentioned reference point. After reaching the desired normal load, the displacement of *s* = 500 µm was established and the disc slid accordingly. The FEM simulations were conducted for four membrane aspect ratios and with a coefficient of friction of 0.3 for each.

## Supplementary information

Below is the link to the electronic supplementary material.

Electronic supplementary material 1 (DOCX 544 kb)
