# Supplementary material for: Variations in strain affect friction and microstructure evolution in copper under a reciprocating tribological load
Source: J Mater Res. 2021 Jan 25;36(4):970–81. doi: 10.1557/s43578-020-00050-z (PMC7610874; doi:10.1557/s43578-020-00050-z)

**Supplementary Information**

**Figure S 1: Schematic of the OFHC sample with two rectangular membranes.**


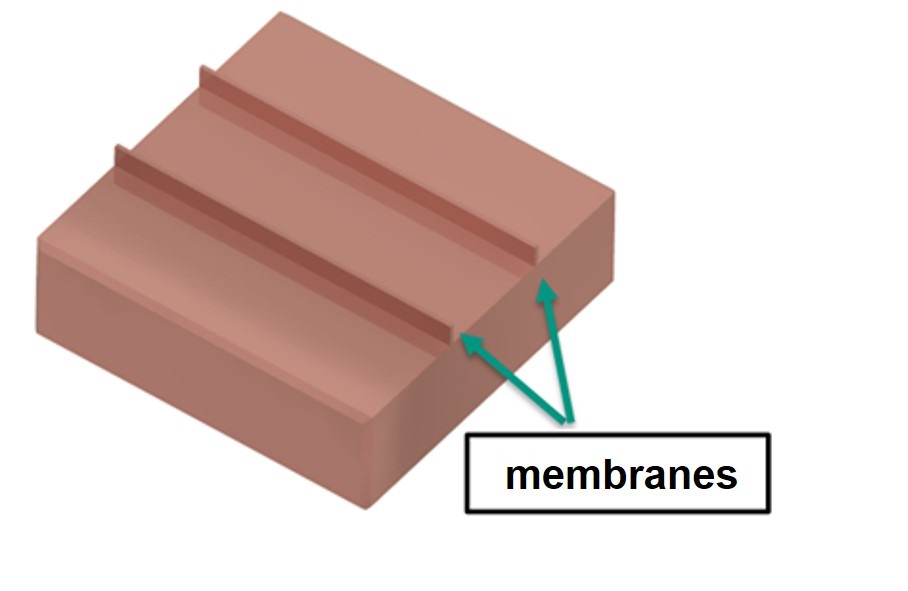


**Figure S 2: FIB cross-sections (a) and (b) and scanning transmission electron microscopy (STEM) images (c) and (d) of membranes before (a)+(c) and after (b)+(d) heat treatment.**

**
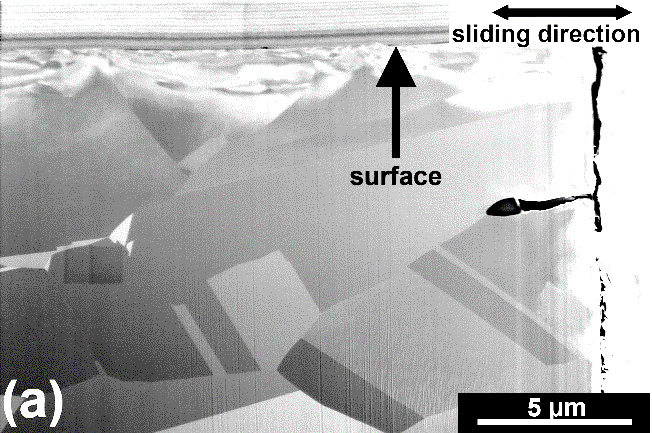

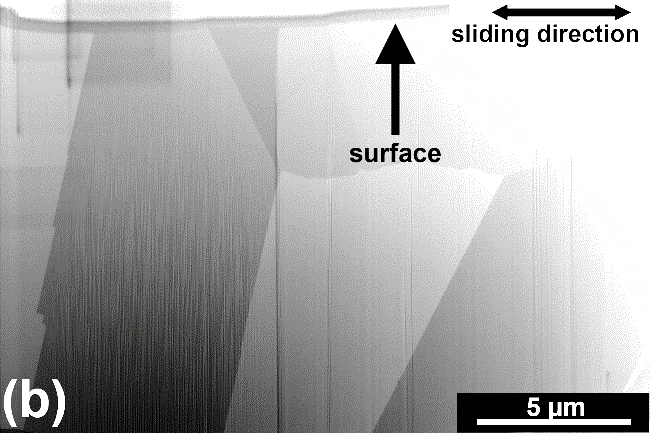
**

**
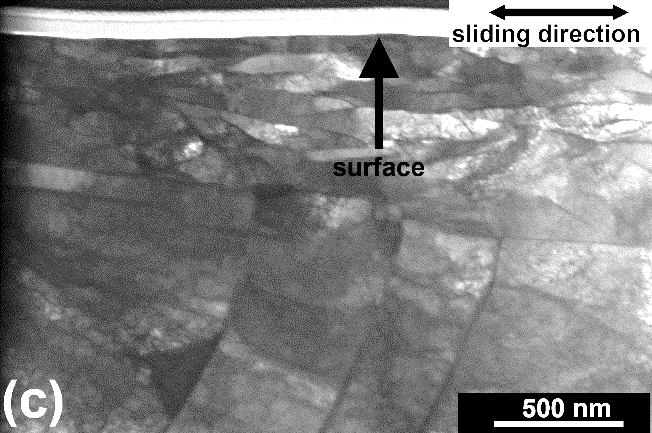

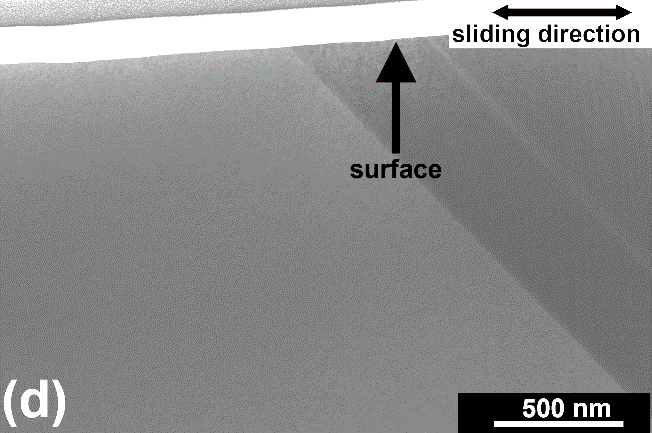
**

**Figure S 3: Schematic of the change in the load configuration due to an increasing aspect ratio.**


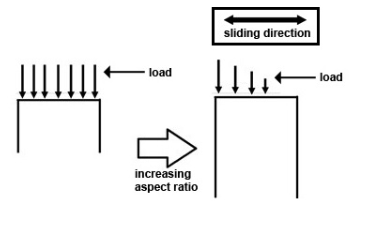


**Figure S 4: Interpretation of the contact behavior of membrane and disc.** Disc and membrane get in contact. The disc is moving from left to right. On the left edge of the membrane a torque occurs. The effective surface is defined as area from the maximum strain value to the position where it becomes zero.


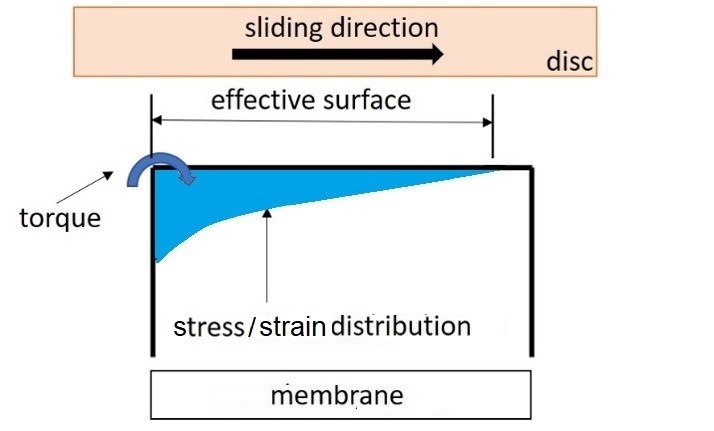

Supplement: Supplementary file 1 — Electronic supplementary material 1 (DOCX 544 kb) [file 43578_2020_50_MOESM1_ESM.docx]
